# Post-pubertal management of undescended testes from the malignancy risk point of view: a systematic review

**DOI:** 10.12688/f1000research.134221.1

**Published:** 2023-09-27

**Authors:** William Tendi, Putu Angga Risky Raharja, Irfan Wahyudi, Arry Rodjani, Gerhard Reinaldi Situmorang

**Affiliations:** 1 Department of Urology, Rumah Sakit Dr Cipto Mangunkusumo, University of Indonesia, Central Jakarta, Jakarta, 10430, Indonesia

**Keywords:** Orchidopexy, Orchiectomy, Testicular malignancy, Undescended testes

## Abstract

**Background:** Undescended testes (UDT) is a condition where one or both testes is absent in the scrotum. The general age recommendation in which the treatment should be performed is before 18 months old due to the infertility risk and malignancy in later life. On the other hand, in post-pubertal UDT, the current guideline recommends orchiectomy; however, this recommendation is weak. Therefore, this study aimed to provide a systematic review of post-pubertal UDT treatment, focusing on the malignancy risk point of view.

**Methods:** A systematic search was performed using PubMed, Wiley Online Library and the Cochrane Library up to 5 March 2023. Any study with either post-pubertal orchiectomy or orchidopexy in patients with UDT and reporting the testicular malignancy was included. The exclusion criteria were studies with lack of information of UDT correction time, no history of correction and the full text wasn’t available. The data collected were the occurrence of testicular malignancy in post-pubertal UDT patients corrected with any method. Quality and bias assessment was assessed with Newcastle-Ottawa scale and Joanna Briggs Institute tools.

**Results:** Seven articles (three case reports and four observational studies) were reviewed with a total of 42 patients who underwent post-pubertal correction of either unilateral or bilateral UDT. The correction age ranged from 13 to 34 years old, with follow-up of 48.7–252 months. Among those who developed malignancies, the most common were seminoma, teratoma and carcinoma in situ of the testes. In addition, this study was able to propose an algorithm for post-pubertal UDT treatment strategy.

**Conclusions:** The scarce resource was the main limitation of this study. Nevertheless, this review showed that post-pubertal UDT management should be tailored individually. Several factors that should be considered include the condition of the contralateral descended testis, UDT location, serum testosterone level, patient’s age, comorbidities, and interest in fertility.

## Introduction

Undescended testes (UDT), or cryptorchidism, is the absence of one or both testes in the scrotum. ^1^ Internationally, the incidence of this condition was 1.0–4.6% of full-term and 1.1–45% of preterm neonates. However, most UDT patients have spontaneous descent in which the prevalence range is between 1–1.5% at the age of three months, 1–2.5% at nine months, and only 1% of all full-term infants still had UDT after reaching one year of age. ^1^^,^^2^

Generally, UDT is classified into palpable and non-palpable, and in most cases, the testis is palpable (80%). The location of the testes is usually within the tract of normal testicular descent, which is the inguinal canal, or far above (intraabdominal). However, it is also possible for the testis to be placed outside the tract (ectopic testis) or not to be present at all, as in the case of agenesis and vanishing testis. Among these types and locations, about 50–60% of non-palpable testis is intraabdominal. ^2^

Regarding the treatment of UDT, the choices of therapy vary between medical management using hormonal therapy and surgical intervention, such as orchidopexy. The current guidelines advise that treatment should start at the age of six months and be finished by 12–18 months to prevent any loss of germ cells and Leydig cells. ^2^ In addition, an early orchidopexy performed before puberty could reduce the risk of testicular germ cell tumour. ^3^

When UDT is not detected or corrected before the patient reaches puberty, there are specific guidelines regarding post-pubertal UDT. The European Association of Urology (EAU) guidelines recommend discussing orchiectomy in such cases with patients. However, this recommendation is weak and seems insufficient. ^4^ Moreover, there is still a possibility of improving the fertility of patients with post-pubertal UDT. Recently, a systematic review conducted by Muncey *et al*. ^5^ revealed that patients with uncorrected bilateral inguinal UDT retain the possibility of fertility after undergoing a post-pubertal orchidopexy. Therefore, adult patients with uncorrected UDT might reconsider orchidopexy instead of orchiectomy. In addition, to the best of the authors’ knowledge, there is no high-level evidence, such as systematic reviews or meta-analyses, regarding the management of post-pubertal UDT from the malignancy risk point of view so far. This study aimed to systematically review the available literature to provide valid recommendations for post-pubertal UDT management, with a focus on the risk of malignancy.

## Methods

### Description of variables and interventions

This study focused on the best option to treat UDT if the condition was not corrected at the age of puberty. Therefore, the population included in this study was male adolescents or adults with a history of UDT who were treated after puberty. If the pubertal status was not explicitly mentioned, the cut-off pubertal age used in this study was 12 years or older, based on an average pubertal age in boys of 11.5 years old. ^6^ The diagnosis of UDT included in this study can either be unilateral or bilateral.

The intervention in this study was orchiectomy, which was compared with orchidopexy. There were no limitations regarding the surgical technique or the approach used in either procedure. In addition, the primary outcome of this study was the occurrence of any testicular malignancy identified after the intervention was performed.

### Literature search and data collection

A comprehensive and systematic literature search was performed on 5 March 2023 in PubMed, Wiley Online Library, and Cochrane Library. The keywords used were “UDT”, “undescended testis”, “undescended testes”, “cryptorchidism”, “orchidectomy”, “orchiectomy”, “testicular removal”, “testes removal”, “testis removal”, “orchidopexy”, “orchiopexy”, “fowler-stephens”, “testicular cancer” and “testicular malignancy”. The study design was either case report, case series, clinical trials or observational studies, such as cohort and case-control. There was no publication time or country restriction. The inclusion criteria in this review were any studies of patients who had a history of UDT and had reached puberty when the orchiectomy or orchidopexy was performed, with a report regarding whether testicular malignancy of any type developed after the surgery, in which there was no limitation in the follow-up time. Studies not written in English were included, as long as the abstract was in English and provided the data needed in this review. The exclusion criteria were studies that did not specify the timing of UDT intervention, had no history of UDT correction at the time of malignancy diagnosis and did not have the full text available online.

All search results were screened for duplicates and relevancy by all authors. After the inclusion and exclusion criteria were applied, the articles included were independently appraised by two reviewers (WT and GRS) and any disagreement was settled by consensus and decided by the third reviewer (PARR). All the searching and screening processes were performed according to the Preferred Reporting Items for Systematic Reviews and Meta-Analyses (PRISMA) flowchart ^7^. The risk of bias assessment and appraisal were performed using the Newcastle–Ottawa scale for cohort and case-control studies. ^8^ The cross-sectional studies and case reports were appraised using the Joanna Briggs Institute (JBI) tool. ^9^ Low quality evidence was determined by a Jadad score of less than three, Newcastle-Ottawa score of less than five, and JBI score of less than 50% (excluded).

### Data extraction

The data collected in this review were the UDT status of the patients (unilateral/bilateral), location of the UDT, age of the patients when the intervention was performed, type of treatment (orchiectomy/orchidopexy), follow-up time, presence of testicular malignancy, type of testicular malignancy and presence of metastasis. Among these variables, the outcome was the occurrence of testicular malignancy. The data extraction, bias and quality assessment were performed by two authors (WT and GRS) and any disagreement was resolved with the third author (PARR). The data extraction, including the assessment of heterogeneity and bias was performed manually and the result was then presented as a table in which all the variables were summarized.

## Results

### Literature search

**Figure 1.**
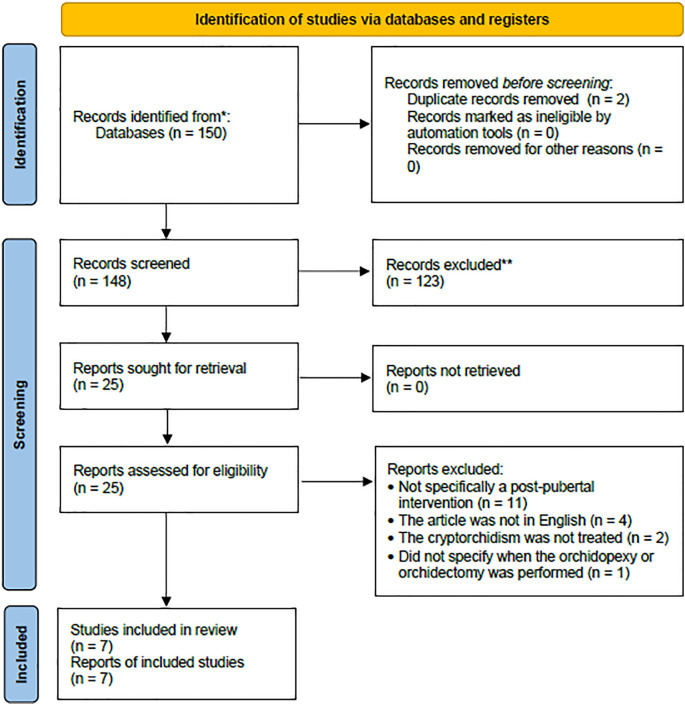
Flowchart of the literature search and screening.

There were initially 150 articles identified, of which there were two duplicates. After screening them for relevancy and applying the inclusion and exclusion criteria, seven studies were included and reviewed ( Figure 1).

### Data extraction and analysis

The risk of bias assessment and appraisal for the seven articles showed that there were three good quality cohort and case-control studies (with a score of 6 for each article), one cross-sectional study and three case reports. All articles presented post-pubertal patient(s) with either unilateral or bilateral UDT and orchidopexy or orchidectomy was performed on the patient(s) with most of the studies observed developed testicular malignancies on later investigation (ranged between 0–252 months after procedure). A summary of the studies’ quality assessment is presented in Table 1. Overall, 42 patients with UDT underwent post-pubertal correction, either by orchidopexy or orchiectomy, which is shown in Table 2. The age of the patients during UDT correction ranged between 13–34 years old and the location of the testes was mostly at inguinal level of intraabdominal. Only one article (Jacobellis *et al*. ^16^) did not report the location of the testes and Lopes *et al*. showed an unusual report of bilateral ectopic testes at the right external inguinal ring and below the spleen. ^15^ In addition, the type of testicular malignancy found in these studies were seminoma, teratoma, carcinoma in situ of the testis, and adenocarcinoma of rete testis. Nevertheless, there was heterogeneity of the outcomes between studies. Torricelli *et al.* found there was no malignancy occurred among the observed 14 patients, and Ford *et al.* reported only one out of 13 post-pubertal unilateral UDT patients corrected with orchidopexy developed malignancy (7.6%). ^10^^,^^11^ However, Wobb *et al*. observed 100% of the patients (3 of 3) had testicular malignancy. ^13^ The heterogeneity might be caused by few numbers of patients observed in both studies. In addition, the mean follow-up time of Torricelli *et al.* was only 48.7 months, and Ford *et al.* did not mention how long the follow-up time was. On the other hand, Wobb *et al.* observed the patients for a mean time of 112 months. Hence, it is possible that the follow-up time might not be long enough for both authors to find proof of testicular malignancy for the other patients.

**Table 1.** Quality assessment of articles included.

Articles	Study design	Quality assessment	
		Newcastle-Ottawa scale	JBI tools
Torricelli FCM, *et al*. (2012) ^10^	Cohort	6	-
Ford TF, *et al*. (1985) ^11^	Cohort	6	-
Pike MC, *et al*. (1986) ^12^	Case-control	6	-
Wobbes TH, *et al*. (1980) ^13^	Cross-sectional	-	Include
Faruk M, *et al.* (2023) ^14^	Case report	-	Include
Lopes RI, *et al*. (2012) ^15^	Case report	-	Include
Jacobellis U, *et al*. (1981) ^16^	Case report	-	Include

JBI=Joanna Briggs Institute.

**Table 2.** Study characteristics.

Articles	Number of patients	Site of UDT	Testis location	Mean or range of patients’ age during correction (years)	Correction history	Time range from UDT correction to cancer investigation (months)	Presence of cancer (n)	Cancer types	Metastasis
Torricelli FCM, *et al*. ^10^	14	Nine unilateral and five bilateral	Intraabdominal	29	13 orchidopexy and six orchiectomies	48.7	No	NA	NR
Ford TF, *et al*. ^11^	13	Unilateral and bilateral	Inguinal or intraabdominal	13–16	Orchidopexy	NR	Yes (1)	Carcinoma in situ	NR
Pike MC, *et al*. ^12^	9	Unilateral and bilateral	Inguinal or intraabdominal	15–34	Orchidopexy or orchiectomy	NR	Yes (NR)	Seminoma or teratoma	NR
Wobbes TH, *et al*. ^13^	3	Unilateral and bilateral	Inguinal or intraabdominal	21.7	Orchidopexy	112	Yes (3)	Seminoma, teratoma or embryonal carcinoma	No
Faruk M, *et al.* ^14^	1	Bilateral	Intraabdominal	24	Right orchiectomy and left orchidopexy	0 (At the same time as the malignancy investigation)	Yes (1)	Seminoma	No
Lopes RI, *et al*. ^15^	1	Bilateral	Right external inguinal ring and below spleen	31	Right orchidopexy	60	Yes (1)	Left carcinoma *in situ*	NR
Jacobellis U, *et al*. ^16^	1	Bilateral	NR	13	Bilateral orchidopexy	252	Yes (1)	Adenocarcinoma of rete testis	Yes, paraaorta lymph node, lungs and mediastinum

NA=not applicable; NR=not reported; UDT=Undescended testes.

Despite the good quality score of the articles included, there were reporting bias of the observational studies, which might contribute to the heterogeneity of the results. The lack of reported follow-up time was also observed in the study conducted by Pike *et al*. Moreover, the authors also did not report specifically how many testicular malignancies occurred among the nine patients observed. Another reporting bias found from most of the observational studies included in this review is the lack of information regarding the specific number of unilateral or bilateral UDT among all the patients. However, the information extracted from the studies included was sufficient to be reviewed to assess the occurrence of testicular malignancy in post-pubertal corrected UDT patients.

## Discussion

The current review showed that most of the patients with post-pubertal UDT had the testes located inguinally or intraabdominally. Only one case from Lopes *et al*. reported a UDT in which the left testis was located just below the spleen. In this unique case, a 36-year-old male presented with infertility and bilateral UDT. The histopathological examination revealed that he had carcinoma *in situ* with splenogonadal fusion in the left testis and no trace of malignancy in his right testis. The patient then underwent a right orchidopexy and a left orchiectomy with the insertion of a testicular prosthesis. ^15^

Regarding the UDT treatment options, the most common post-pubertal procedure performed in this study was orchidopexy. The study by Wobbes *et al*. revealed that all three patients who underwent an orchidopexy after puberty developed a testicular tumour in 112 months (mean time range) after orchidopexy, regardless of whether the UDT was unilateral or bilateral. The histopathological examination showed seminoma and embryonal carcinoma in two patients with right unilateral UDT and teratoma/seminoma in the left testis of a bilateral UDT patient who underwent bilateral orchidopexy. Interestingly, the study also observed that nine other patients underwent orchidopexy just before puberty and all developed testicular malignancy in 12–29 years after intervention. This fact suggests that late orchidopexy, both before and after puberty, might not reduce the malignancy risk well. ^13^

However, one article revealed that no malignancy occurred in patients who underwent post-pubertal intervention. Torricelli *et al*. assessed 14 unilateral and bilateral UDT patients with a mean age of 29 years (18–54 years) when the UDT was corrected with either orchidopexy or orchiectomy. All of the testes were located intraabdominally, and the orchiectomies were performed due to either the small size or difficulty in dissection. The mean follow-up time was 48.7 months, and there was no malignancy found in any of the patients, despite the higher chance of carcinoma *in situ* in intraabdominal UDT. This suggests the same level of efficacy of both orchidopexy and orchiectomy in a post-pubertal setting. ^3^^,^^10^ Nevertheless, it should also be noted that the follow-up time was the shortest among the reported times in this review.

In addition, a study by Ford *et al*. showed that of all 13 unilateral and bilateral UDT patients who had their testes located either inguinally or intraabdominally and underwent post-pubertal orchidopexy, only one developed carcinoma *in situ.* This suggests that orchidopexy might still be a valid choice in treating post-pubertal UDT. The tumour occurred on the contralateral normal descended testis of one of the unilateral UDT patients. ^11^

Pike *et al*. observed that nine patients with unilateral and bilateral UDT underwent either orchidopexy or orchiectomy at the age of 15–34 years. The study revealed that there was still a chance of testicular malignancy after correction. However, the authors stated that the age distribution of UDT correction, both pre- and post-pubertal, was the same as the expected rate of the national population, suggesting that there was no association between the time of correction and the risk of testicular malignancy. Unfortunately, the study did not specifically mention which treatment group developed malignancy. ^12^

### Post-pubertal recommendation: take a chance on fertility, or risk malignancy?

As mentioned above, a recent systematic review showed that there was a possibility of fertility in men with UDT after orchidopexy, even at an age as late as 38 years old. The parameters used in determining fertility status were sperm count and the evaluation of hormones, including testosterone. However, the authors of that study also mentioned that in bilateral UDT cases in which the testes were located intraabdominally, germ cells were not found. ^5^ A possible explanation of this phenomenon was the non-ideal temperature of the testes for the germ cells to grow. ^3^

A similar result was presented by Rohayem *et al*. The study stated that despite the inverse correlation among the age of UDT correction, testicular volume and sperm concentration, there were patients who had a late correction and still had normozoospermia and normal serum testosterone while some who had an early correction had azoospermia. Therefore, the chance of UDT patients having a biological child is not lost, even when the condition was neglected after puberty. ^17^

On the other hand, the risk of testicular germ cell tumours increased five- to 10-fold in patients with UDT compared with the general population and even more so when the UDT was not corrected until puberty and thus post-pubertal orchiectomy was recommended. ^3^^,^^18^ However, a recent study by Xu *et al*. suggested that the recommendation of orchiectomy might depend on the location of the UDT in which orchiectomy or testicular biopsy for malignancy screening in older boys or adolescents should be considered in intraabdominal UDT, whereas inguinal UDT did not mandate the need of testicular biopsy at the time of orchidopexy. In this study, a total of 71 patients aged 10.1–27.7 years old were included and two of the patients developed testicular malignancy (age 16.2 and 26.6 years old). Both patients had intraabdominal UDT and underwent unilateral orchiectomy in which the histopathological finding revealed seminomas. ^19^ The most recent report in January 2023 was published by Faruk *et al*., in which the authors found a right testicular seminoma in a 24-year-old man with uncorrected bilateral UDT. In the study, the patient underwent right orchiectomy and left orchidopexy followed by close monitoring every four months for the first year, every six months for the second year and then annually for the third to fifth years. ^14^

**Figure 2.**
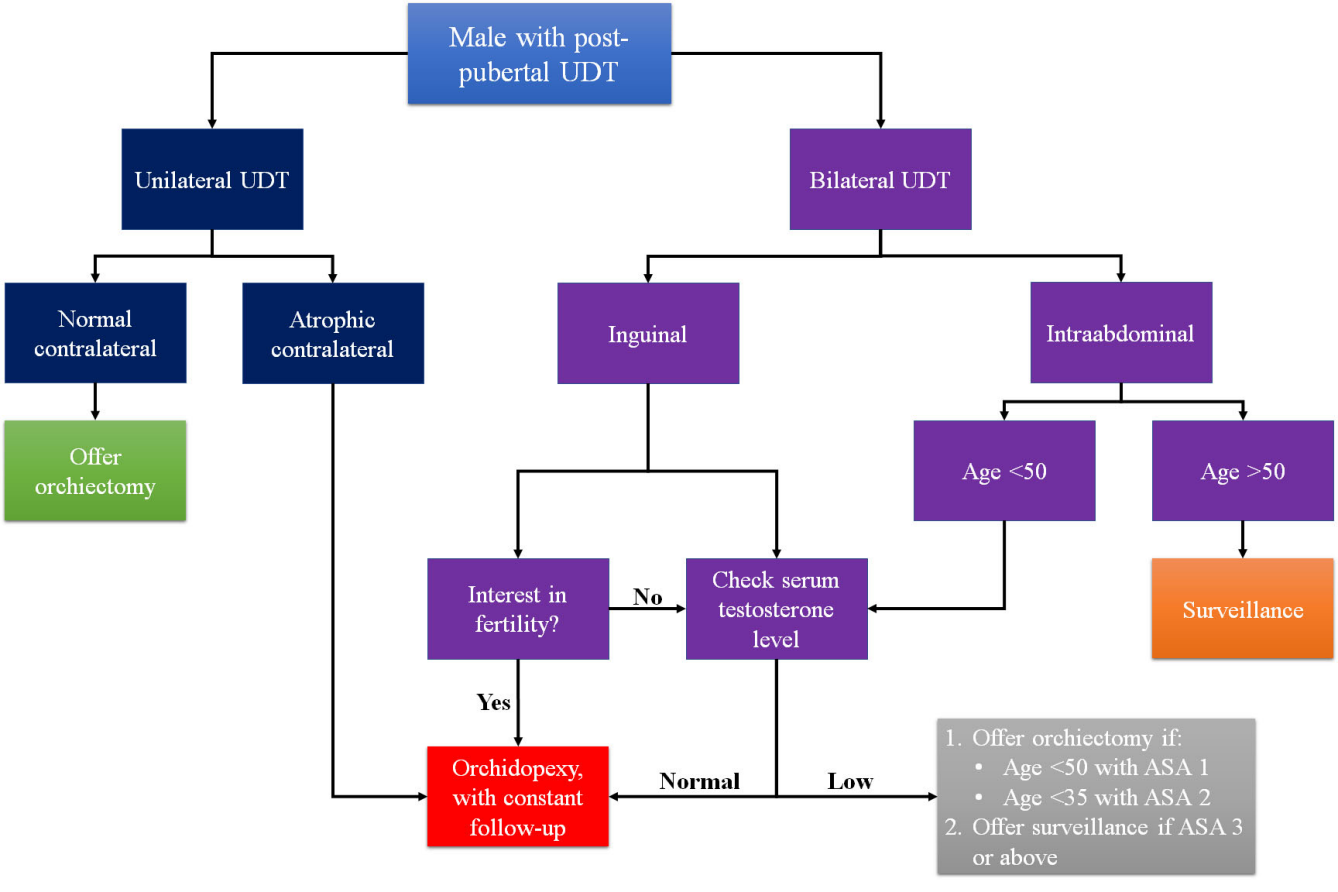
Proposed algorithm of post-pubertal UDT. UDT=undescended testes.

In addition, a mortality risk analysis of post-pubertal UDT was published recently by Shah *et al*. The study balanced the risks of perioperative mortality (POM) and death from germ cell tumours based on age group when the UDT was diagnosed. The POM was defined based on the criteria determined by the American Society of Anesthesiologists (ASA). The result of this study was that prophylactic orchiectomy should only be considered when the patients’ age was below 50 years and healthy (ASA class 1). However, if the patients had mild systemic diseases (ASA class 2), the procedure could be considered when the patients’ age was below 35 years. Patients with ASA class 3 or above should always undergo observation. ^20^ Based on these studies, we propose an algorithm regarding the recommendation of post-pubertal UDT management ( Figure 2).

### Important notes in late UDT treatment

After either an orchiectomy or orchidopexy, patients should be advised to routinely perform a self-examination, especially those with unilateral correction. In unilateral UDT, there was a slight increase in the risk of malignancy of the contralateral testis. ^3^ A case of contralateral testicular malignancy in unilateral UDT was described by Ueda *et al*. in 1995. The study showed the case of a 36-year-old man who had left unilateral UDT and presented with painless swelling of his right scrotum. Further examination revealed a seminoma on the right testis, which was then removed. The left testis was found in the left inguinal region, and a left orchiectomy was eventually performed. However, in this case the removal of the left testis was performed because of infection instead of malignancy since the histopathological examination of the left testis revealed only atrophy of germ cells without any atypia of either Leydig cells or germ cells. ^21^

Another consideration regarding post-pubertal treatment is the presence of testicular torsion. The incidence of testicular torsion in uncorrected UDT is higher, which was proved by a case presented by McDermott *et al*. The authors described a 42-year-old man who had a bilateral UDT and a history of right orchidopexy and came with abdominal peritonism. Further investigation revealed a torted seminomatous testicular tumour from the previously neglected left testis. ^3^^,^^22^ A study by Naouar *et al*. showed that adult patients with UDT and testicular torsion were managed based on the intraoperative finding of the testes. Orchiectomy was preferred when there was a mass or testicular necrosis, while orchidopexy was still an available choice for simple torsion. ^23^

In addition, a study by Yuksel *et al*. described the possibility of persistent Müllerian duct syndrome (PMDS) in bilateral UDT. The article showed a 26-year-old male who presented with infertility and swelling of his right inguinal region. Further examination revealed a right inguinal hernia with bilateral UDT. The patient was then scheduled for a laparoscopic exploration, in which his right testis was found attached to a uterus by a round ligament and tuba uterina and his left testis was found atrophic and attached to the other side of the uterus. The left testis was then removed and a right orchidopexy was performed along with a right inguinal herniography. The presence of the uterus and bilateral tuba uterina was suggestive of PMDS, and a histopathological examination of his right testis revealed an intratubullary germ cell neoplasia. ^24^

The possibility of PMDS in UDT was important because it could develop into a Müllerian malignancy and be fatal. A study by Farikullah *et al*. showed the importance of Müllerian remnant removal when performing orchidopexy. The study observed eight patients in a series that had PMDS and UDT. One of the patients was a 44-year-old male who had bilateral UDT and underwent bilateral inguinal exploration and a left orchidopexy at the age of 18. However, the Müllerian structure in this patient was not seen at the laparoscopy, resulting in a missed PMDS. The patient was then diagnosed with squamous cell carcinoma of the Müllerian duct 26 years later and died of metastasis. ^25^ Therefore, a thorough investigation of Müllerian structure is important when performing orchidopexy, especially in the case of bilateral UDT, and any Müllerian remnant found during examination should be removed.

The limitation of this study is that the resources regarding post-pubertal treatment in UDT were scarce and the only articles available were case reports and observational studies with few research subjects, resulting in heterogeneity between studies. In addition, most of the articles were outdated and did not mention the pubertal status of the patients; thus, we could only assume the post-pubertal status based on the common age of male puberty, as described in the methods section. Nevertheless, this is the first systematic review providing treatment recommendations in post-pubertal UDT by considering the malignancy risk.

## Conclusions

To conclude, in terms of malignancy risk, several factors should be considered when deciding post-pubertal UDT treatment, including the age of the patients, comorbidity of the patients, interest in fertility, and location of the testes. The authors recommend performing orchiectomy in post-pubertal unilateral UDT patients with normal contralateral testes, post-pubertal inguinal bilateral UDT patients that had no interest in fertility, and post-pubertal intraabdominal bilateral UDT patients aged less than 50 with no comorbidity or less than 35 with mild systemic disease. Otherwise, surveillance in patients older than 50 years old and orchidopexy in younger patients could still be considered. Therefore, it is important to clearly explain both the risks and possible benefits of both orchidopexy and orchiectomy to all post-pubertal UDT patients.

## Data Availability

All data underlying the results are available as part of the article and no additional source data are required. Open Science Framework: PRISMA checklist for article ‘Supine versus prone position in percutaneous nephrolithotomy: a systematic review and meta-analysis’, https://doi.org/10.17605/OSF.IO/K5QCJ. ^7^ Data are available under the terms of the Creative Commons Zero “No rights reserved” data waiver (CC0 1.0 Public domain dedication).
