# Supplementary material for: Post-pubertal management of undescended testes from the malignancy risk point of view: a systematic review
Source: F1000Res. 2024 Nov 13;12:1226. Originally published 2023 Sep 27. [Version 3] doi: 10.12688/f1000research.134221.3 (PMC11470230; doi:10.12688/f1000research.134221.3)
Supplement: Peer review history

# Reviewer report: Reviewer response for version 3

**DOI:** 10.5256/f1000research.173401.r340316

**Published:** 2024-11-19

**Authors:** Ludovic Dumont

**Affiliations:** 1 Univ Rouen Normandie, INSERM, UMR, France

In their Systematic Review *“Post-pubertal management of undescended testes from the malignancy risk point of view: a systematic review”*, the authors selected seven articles (three good quality cohort and case-control studies, one cross-sectional study, and three case reports) with a total of 42 patients who underwent post-pubertal correction of either unilateral or bilateral undescended testes. The study is well done, systematic, intelligible and the methodology (with presentation of PRISMA Flowchart, the use of JBI and Jadad Score, *etc*.) highly appreciated and clearly explained.

Are the rationale for, and objectives of, the Systematic Review clearly stated?

Yes

Is the statistical analysis and its interpretation appropriate?

Not applicable

If this is a Living Systematic Review, is the ‘living’ method appropriate and is the search schedule clearly defined and justified? (‘Living Systematic Review’ or a variation of this term should be included in the title.)

Not applicable

Are sufficient details of the methods and analysis provided to allow replication by others?

Yes

Are the conclusions drawn adequately supported by the results presented in the review?

Yes

Reviewer Expertise:

Biomedical Engineering Developmental Biology Cryobiology Histology Spermatogonial Stem Cell Spermatogenesis Gametogenesis Cryopreservation Oocyte Culture Vitrification Micromanipulation In Vitro Culture Conventional PCR Histological Analysis Fluorescence Microscopy Reproductive Biology Histology Cryobiology Endocrinology single cell Reproduction Biology RNA-Seq

I confirm that I have read this submission and believe that I have an appropriate level of expertise to confirm that it is of an acceptable scientific standard.

# Reviewer report: Reviewer response for version 2

**DOI:** 10.5256/f1000research.168034.r327173

**Published:** 2024-10-18

**Authors:** Rifat Burak Ergül

**Affiliations:** 1 Istanbul University, Istanbul, Turkey

1. March 5, 2023 is approximately 1.5 years ago from now. I recommend that the authors conduct a new, updated literature search. This will result in a more current publication. (For example, a related publication in 2024 is available with the Ergül R, et al., 2024 [Ref-2]
2. I don't understand why the researchers did not include a key article on this topic, such as 'Koni A, et al., 2024 ^1^,' in their study. When I review the inclusion criteria of the paper, it seems to meet them. Based on the inclusion and exclusion criteria, the literature review should be reassessed.
3. While addressing this topic, it is also necessary to discuss Testicular Dysgenesis Syndrome. It is recommended that the researchers add a paragraph on this topic in the discussion section.

Are the rationale for, and objectives of, the Systematic Review clearly stated?

Yes

Is the statistical analysis and its interpretation appropriate?

Yes

If this is a Living Systematic Review, is the ‘living’ method appropriate and is the search schedule clearly defined and justified? (‘Living Systematic Review’ or a variation of this term should be included in the title.)

Not applicable

Are sufficient details of the methods and analysis provided to allow replication by others?

Partly

Are the conclusions drawn adequately supported by the results presented in the review?

Yes

Reviewer Expertise:

Cryptorchidisim, Endo-urology- Stone- PCNL-URS- Prostate Cancer

I confirm that I have read this submission and believe that I have an appropriate level of expertise to confirm that it is of an acceptable scientific standard, however I have significant reservations, as outlined above.

## References

[1] : Histopathological Evaluation of Orchiectomy Specimens in 51 Late Postpubertal Men with Unilateral Cryptorchidism. *Journal of Urology* .2014;192(4) : 10.1016/j.juro.2014.05.048 1183-1188 10.1016/j.juro.2014.05.048 24840535

[2] : Risk for testicular germ cell tumors and spermatogenesis failure in post-pubertal undescended testes. *International Urology and Nephrology* .2024;56(7) : 10.1007/s11255-024-03963-4 2269-2274 10.1007/s11255-024-03963-4 38349599

# Reviewer report: Reviewer response for version 2

**DOI:** 10.5256/f1000research.168034.r327171

**Published:** 2024-10-11

**Authors:** Ludovic Dumont

**Affiliations:** 1 Univ Rouen Normandie, INSERM, UMR, France

In their Systematic Review *“Post-pubertal management of undescended testes from the malignancy risk point of view: a systematic review”*, the authors selected seven articles (three good quality cohort and case-control studies, one cross-sectional study, and three case reports) with a total of 42 patients who underwent post-pubertal correction of either unilateral or bilateral undescended testes. The study is well done, systematic, intelligible and the methodology (with presentation of PRISMA Flowchart, the use of JBI and Jadad Score, *etc*.) highly appreciated and clearly explained. However, there are some changes or proposals for corrections that have to be addressed.

 General Comments

 • Please find a system for numbering the lines (at least a version for reviewers) as it is difficult to target the precise changes to be made to the text.

 Specific to the Figures and Table

 • The PRISMA flowchart shows asterisks (* and **), but nowhere does it seem to indicate what they correspond to.

 Specific Comments

 • Page 1 in the Background. Please, rewrite the sentence *“On the other hand, in postpubertal UDT, the current guideline recommends orchiectomy; however, this recommendation is weak.”*.

 • Page 2. Please, italicize “in situ” throughout the entire manuscript.

 • Page 3. Is it necessary to specify details like *“to the best of the author's knowledge”*?

Are the rationale for, and objectives of, the Systematic Review clearly stated?

Yes

Is the statistical analysis and its interpretation appropriate?

Not applicable

If this is a Living Systematic Review, is the ‘living’ method appropriate and is the search schedule clearly defined and justified? (‘Living Systematic Review’ or a variation of this term should be included in the title.)

Not applicable

Are sufficient details of the methods and analysis provided to allow replication by others?

Yes

Are the conclusions drawn adequately supported by the results presented in the review?

Yes

Reviewer Expertise:

Biomedical Engineering Developmental Biology Cryobiology Histology Spermatogonial Stem Cell Spermatogenesis Gametogenesis Cryopreservation Oocyte Culture Vitrification Micromanipulation In Vitro Culture Conventional PCR Histological Analysis Fluorescence Microscopy Reproductive Biology Histology Cryobiology Endocrinology single cell Reproduction Biology RNA-Seq

I confirm that I have read this submission and believe that I have an appropriate level of expertise to confirm that it is of an acceptable scientific standard.

# Response

**Published:** 2024-10-12

**Authors:** William Tendi

**Affiliations:** Department of Urology, Dr. Cipto Mangunkusumo Hospital, Jakarta, Indonesia

1. Thank you for your kind review. We have added a line number for the next version of the article submitted

 2. Thank you for your kind review. The asterisks were from the original template of the PRISMA guideline from the website, meaning to guide the authors about how to fill the boxes from the template. We have removed the asterisks

 3. Thank you for your kind comment, we have revised the sentence accordingly

 4. Thank you for our kind comment, we have revised the manuscript accordingly

 5. The sentence "to the best of the author's knowledge" means to specify that prior to the publication of the manuscript, there has been no systematic review or meta-analysis that provide the evidence or specific algorithm of the post-pubertal UDT treatment, at least to the best of our search in the various databases included in the study. Nevertheless, we have removed the sentence. Thank you for your kind comment

# Reviewer report: Reviewer response for version 2

**DOI:** 10.5256/f1000research.168034.r293772

**Published:** 2024-06-24

**Authors:** Biagio Barone

**Affiliations:** 1 AORN Sant’Anna e San Sebastiano, Caserta, Italy

The authors improved the manuscript according to previous suggestions. No further corrections required.

Are the rationale for, and objectives of, the Systematic Review clearly stated?

Yes

Is the statistical analysis and its interpretation appropriate?

Yes

Are sufficient details of the methods and analysis provided to allow replication by others?

Yes

Are the conclusions drawn adequately supported by the results presented in the review?

Yes

Reviewer Expertise:

Urology and Andrology

I confirm that I have read this submission and believe that I have an appropriate level of expertise to confirm that it is of an acceptable scientific standard.

# Response

**Published:** 2024-10-12

**Authors:** William Tendi

**Affiliations:** Department of Urology, Dr. Cipto Mangunkusumo Hospital, Jakarta, Indonesia

Thank you for your kind review

# Reviewer report: Reviewer response for version 1

**DOI:** 10.5256/f1000research.147257.r252570

**Published:** 2024-03-25

**Authors:** Biagio Barone

**Affiliations:** 1 AORN Sant’Anna e San Sebastiano, Caserta, Italy

General comment

 The manuscript entitled "A Systematic Review on Post-Pubertal Management of Undescended Testes: Focus on Malignancy Risk” presents a systematic review on the management of undescended testes in post-pubertal individuals, with a focus on the risk of malignancy. The manuscript is well-structured and addresses an important clinical issue. However, there are areas for improvement in clarity, methodology, and interpretation of results.

- While the manuscript generally provides clear descriptions of methods and results, some sections could benefit from improved clarity and organization. Check grammar and sentences along the text.
- The authors appropriately acknowledge heterogeneity among included studies but should further discuss potential sources of heterogeneity and its impact on the synthesis of findings.
- About the risk of malignancy related to undescended testes, also see: [1]
- The review should explicitly address its limitations, such as its reliance on observational studies and the potential publication bias of studies reported. This will ensure a balanced interpretation of the evidence.
- The recommendations provided in the conclusion should be supported by a more robust synthesis of evidence, including consideration of potential biases and uncertainties.

Are the rationale for, and objectives of, the Systematic Review clearly stated?

Yes

Is the statistical analysis and its interpretation appropriate?

Yes

Are sufficient details of the methods and analysis provided to allow replication by others?

Yes

Are the conclusions drawn adequately supported by the results presented in the review?

Yes

Reviewer Expertise:

Urology and Andrology

I confirm that I have read this submission and believe that I have an appropriate level of expertise to confirm that it is of an acceptable scientific standard, however I have significant reservations, as outlined above.

## References

[1] : Risk Factors for Testicular Cancer: Environment, Genes and Infections-Is It All?. *Medicina (Kaunas)* .2023;59(4) : 10.3390/medicina59040724 10.3390/medicina59040724 37109682 PMC10145700

# Response

**Published:** 2024-06-17

**Authors:** William Tendi

**Affiliations:** Department of Urology, Dr. Cipto Mangunkusumo Hospital, Jakarta, Indonesia

1. Thank you for your kind review. We have revised the manuscript accordingly especially in the Introduction and Discussion sections

 2. Thank you for your kind review. We have discussed further the heterogeneity on the Discussion section, paragraph 15 and Conclusion section

 3. Thank you for your kind suggestion. We have added and cited additional information from the suggested articles at the Discussion section, paragraph 9, reference number 20

 4. Thank you for your review. We have discussed the limitation of the study and potential bias in the Discussion section, paragraph 15-16

 5. Thank you for your review. We have revised the Conclusion section accordingly
